# Forecasting individual progression trajectories in Huntington disease enables more powered clinical trials

**DOI:** 10.1038/s41598-022-18848-8

**Published:** 2022-11-07

**Authors:** Igor Koval, Thomas Dighiero-Brecht, Allan J. Tobin, Sarah J. Tabrizi, Rachael I. Scahill, Sophie Tezenas du Montcel, Stanley Durrleman, Alexandra Durr

**Affiliations:** 1 Institut du Cerveau - Paris Brain Institute - ICM, CNRS, Inria, Inserm, AP-HP, Hôpital de la Pitié Salpêtrière, Sorbonne Université, 75013 Paris, France; 2 Biological Adaptation and Ageing, Sorbonne Université, Paris, France; 3 Brain Research Institute, University of California Los Angeles, Los Angeles, CA USA; 4 UCL Queen Square Institute of Neurology, University College London, Queen Square, London, UK; 5 Department of Neurology, DMU Neurosciences, Sorbonne Université, Institut du Cerveau - Paris Brain Institute - ICM, CNRS, Inserm, AP-HP, Hôpital de la Pitié Salpêtrière, 75013 Paris, France

**Keywords:** Computational models, Huntington's disease, Prognostic markers, Statistics, Clinical trial design

## Abstract

Variability in neurodegenerative disease progression poses great challenges for the evaluation of potential treatments. Identifying the persons who will experience significant progression in the short term is key for the implementation of trials with smaller sample sizes. We apply here disease course mapping to forecast biomarker progression for individual carriers of the pathological CAG repeat expansions responsible for Huntington disease. We used data from two longitudinal studies (TRACK-HD and TRACK-ON) to synchronize temporal progression of 15 clinical and imaging biomarkers from 290 participants with Huntington disease. We used then the resulting HD COURSE MAP to forecast clinical endpoints from the baseline data of 11,510 participants from ENROLL-HD, an external validation cohort. We used such forecasts to select participants at risk for progression and compute the power of trials for such an enriched population. HD COURSE MAP forecasts biomarkers 5 years after the baseline measures with a maximum mean absolute error of 10 points for the total motor score and 2.15 for the total functional capacity. This allowed reducing sample sizes in trial up to 50% including participants with a higher risk for progression ensuring a more homogeneous group of participants.

## Introduction

One recent phase-III clinical trial failed to show that carefully selected antisense oligonucleotides halted the progression of Huntington disease (HD)^1,2^. This failure was disappointing for the whole community and particularly for the more than 800 early-stage participants. Future trials—both for dosing (phase II) and for efficacy (phase III)—should attempt to reach conclusion with as few participants as possible. Sample size of proof-of-concept phase II trials is important to drug development in general.

The ability to detect treatment-dependent improvements in a clinical trial depends on the ability to assess the risk of significant outcome changes. The motivation, here, is simple: treating a patient for whom the selected endpoint is likely to display little natural progression during the trial cannot provide evidence about the possibility of a candidate therapy changing the outcome. Patients should be observed over a period during which the selected endpoint is expected to worsen significantly without intervention. The target period depends on the endpoint chosen for the demonstration of efficacy.

Individual variability impedes prognosis and prediction of multiple possible endpoints in progressive disorder. Even for HD, whose molecular aetiology is well known, we cannot predict the course of disease. Broadly, a higher number of CAG repeats within the HTT gene predicts earlier onset, but two people with the same repeat length may differ in clinical onset by decades^3–6^. This variability poses a serious challenge for the evaluation of potential treatments, particularly in early stages.

Several groups reported methods for risk assessment and subject selection in trials. These methods initially relied only on age and repeat length, while more recent analyses have also included motor and cognitive assessments. The published indices include (1) “disease burden” or the CAG repeat-Age Product (CAP), (2) CAG repeat-Age Product Scaled (CAPS), (3) Prognostic Index (PIN), which includes age and repeat length plus motor and cognitive assessments, and (4) multivariate risk score (MRS), which incorporates age, repeat length, motor and cognitive assessments, and diagnostic confidence level^7–10^. CAP and CAPS account only for age and repeat length as factors determining the disease onset. PIN increases sensitivity to treatment effects for several clinical endpoints besides disease onset^11^. This property of PIN is only incidental, however, since PIN’s purpose was to predict clinical onset, not the progression of clinical or biological markers across all stages. Extension to such markers would require the calculation and validation for each index. The Multivariate Risk Score (MRS) can distinguish the gene carriers who are most likely to develop the disease within 3 years^12^.

We propose here to use a disease modeling technique, called disease course mapping, to enrich the trial population in participants likely to exhibit progression of a given endpoint during trial. Disease course mapping charts the range of possible disease trajectories described by a series of endpoints and biomarkers. The method uniquely accounts for variations in the dynamics of progression, distinguishing age at onset and pace of progression, and in disease presentation, distinguishing the specific timing and ordering of each biomarker relatively to the other ones. We have developed this statistical learning technique, and applied it to neurodegenerative diseases^13^. In particular, we showed that this technique predicts cognitive decline with 20% better accuracy than 56 alternative methods in Alzheimer’s disease^14^.

We used here two multicenter longitudinal studies—TRACK-HD and TRACK-ON—to learn HD COURSE MAP, the disease progression model describing the progression of a series of imaging biomarkers and clinical endpoints. Then, we used HD COURSE MAP to forecast the progression of endpoints from the baseline measurements of each HD-pathological expansion carriers in an independent data set: ENROLL-HD. We assessed the ability of these forecasts to accurately identify the participants whose selected endpoint experienced a significant progression during a given period. With the same model, we could seamlessly evaluate several endpoints and trial duration at various disease stages. We therefore simulated several hypothetical trials by varying outcome, duration and disease stage. We computed sample sizes using our enriched population and compared them with standard inclusion criteria and enrichment based on CAP and PIN.

## Results

We used data from 290 participants in TRACK-HD and TRACK-ON studies (1413 visits). TRACK-HD followed 131 pre-symptomatic HD-mutation carriers, 126 patients with early-stage HD and 4 with an unknown status at baseline, to which we added 29 pre-symptomatic HD-mutation carriers from the follow-up study TRACK-ON. These studies also included 186 “control” subjects, most of whom were relatives without a pathogenic HD-mutation. We used data of 11,510 participants from ENROLL-HD with a total number of 28,189 visits as external validation set (see Table 1).

**Table 1:** Demographic description of participants from the two TRACK studies (TRACK-HD and TRACK ON) and the ENROLL-HD study.

Variable description and characteristics	TRACK HD & TRACK ON	ENROLL
Number of participants (and of visits)	290 (1413)	11,510 (28,189)
Distributions of follow-up visits	4 participants with 1 visit, 3 with 2 visits, 22 with 3 visits, 159 with 4 visits, 4 with 5 visits, 5 with 6 visits, 93 with 7 visits	3599 participants with 1 visit, 2764 with 2 visits, 2535 with 3 visits, 1777 with 4 visits, 665 with 5 visits, 166 with 6 visits4 with 7 visits
Age (± std) years old [Min; Max]	45.3 (± 10.1) [18.6; 70.2]	49.0 (± 13.8) [18.0; 92.0]
Number of CAG repetitions (± std) [Min; Max]	43.4 (± 2.7) [39; 59]	43.6 (± 3.6) [37; 70]
Women/men/unknown (%)	54.1/44.5/1.4	53.9/46.1/0
Education level (± std) [Min; Max]	Number of years: 3.8 (± 1.2) [1; 6]	ISCED level: 3.6 (± 1.2) [0; 6]
Clinical features	Total motor score from the unified huntington disease rating scale (TMS) Total functional capacity from the UHDRS (TFC), and the apathy rating from the problem behavior assessment (PBA-Apathy)	Total motor score from the unified huntington disease rating scale (TMS) Total functional capacity from the UHDRS (TFC), and the apathy rating from the problem behavior assessment (PBA-Apathy)
Cognitive features	Stroop word reading test (Stroop), symbol digit modalities test (SDMT), direct circle tracing (Circle Tracing), and indirect circle tracing (circle tracing indirect)	Stroop word reading test (Stroop), and symbol digit modalities test (SDMT)
Imaging features (volumes)	(1) Striatum, (2) Globus pallidus, (3) Putamen, (4) Caudate nucleus, (5) White matter, (6) Grey matter, (7) Ventricles and total brain	–

We selected 15 features in our initial modelling of biomarkers’ progression based on TRACK-HD and TRACK-ON participants:

- Clinical (1) TMS, the total motor score from the Unified Huntington disease Rating Scale [UHDRS], (2) Total Functional Capacity [TFC] from the UHDRS and (3) the apathy rating from the Problem Behavior Assessment [PBA-Apathy].
- Cognitive (1) Stroop Word Reading Test [Stroop], (2) Symbol Digit Modalities Test [SDMT], (3) Direct Circle Tracing, and (4) Indirect Circle Tracing.
- Imaging (1) striatum, (2) globus pallidus, (3) putamen, (4) ventricles, (5) caudate nucleus, (6) white matter, (7) grey matter, and (8) total brain volumes– all normalized to total intracranial volume.

### HD COURSE MAP summarizes the progression of 15 clinical and radiological outcomes across all disease stages

**Figure 1:**
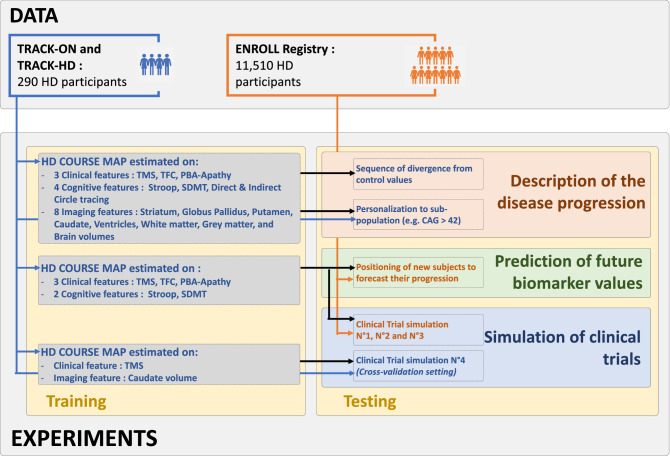
HD COURSE MAP is trained on 290 participants from TRACK studies and tested on independent 11,510 participants from ENROLL-HD. Description of disease progression is based on HD COURSE MAP calibrated on 15 biomarkers. Forecasts of biomarker progression and selection in trials are based on HD COURSE MAP calibrated on the 5 biomarkers that are present in both TRACK and ENROLL. Forecasts of imaging biomarkers are validated on TRACK data only using a cross-validation procedure.

We first established HD COURSE MAP from the 290 participants from TRACK-HD and TRACK-ON studies that were followed up to 6.6 years. Subjects included both asymptomatic and symptomatic carriers of the pathological expansion (see Fig. 1).

**Figure 2:**
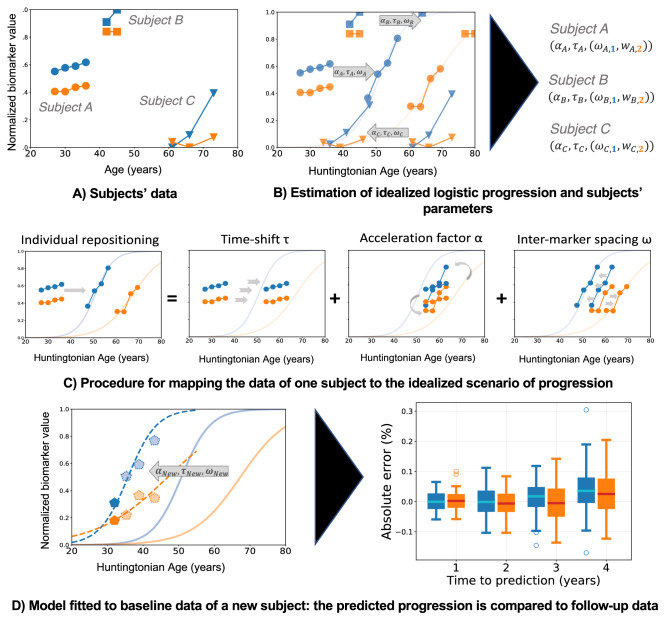
HD COURSE MAP describes the changes of clinical and imaging biomarkers in early HD; and then predicts the future values of these biomarkers at the entry of a new participant. (**A**) Schematic representation of two imagined biomarkers (orange and blue) for three subjects of different ages and disease stages (Subject A, B and C). Biomarker values are normalized between 0 and + 1, the latter corresponding to the maximum pathological value. Example: subject C shows biomarker values similar to controls at age 60, but the blue biomarker increased to 0.4 at age 72. (**B**) The method estimates two logistic curves for the two biomarkers. They depict the progression of the typical subject in the population and are indexed by HA, a disease stage. These curves are estimated together with the parameters transforming the curves to best fit each biomarker and each subject. (**C**) Three parameters describe the relationship of the logistic curves to the actual data: α (acceleration factor); τ (time shift), and ω (intermarker spacing). The modeling assumes that each subject has a single α, a single τ, and an ω for each biomarker. The method uniquely decomposes differences in dynamics of progression (acceleration factor and time-shift) and disease presentation at the same HA (intermarker spacing) (**D**) The curves are fitted to the baseline data a new subject. The best-fit parameters predict a personalized trajectory of changes of the subject’s biomarkers. The predicted values are compared to the hidden subject’s data at follow-up visits 1–4 years after baseline.

HD COURSE MAP summarizes the long-term trajectory of clinical evaluations, cognitive assessments and radiological volumes along a normalized time axis called “Huntingtonian Age “(HA). HA allows to position the patient’s visits at heterogeneous stages onto a unique time axis which then serve as a disease-staging scale. The model maps the chronological age of each participant at each visit to one HA (see Fig. 2 and “Methods” section for details). Participants here had HAs between 30 and 80 years, so our model represents about 50 years of the natural history of the disease.

**Figure 3:**
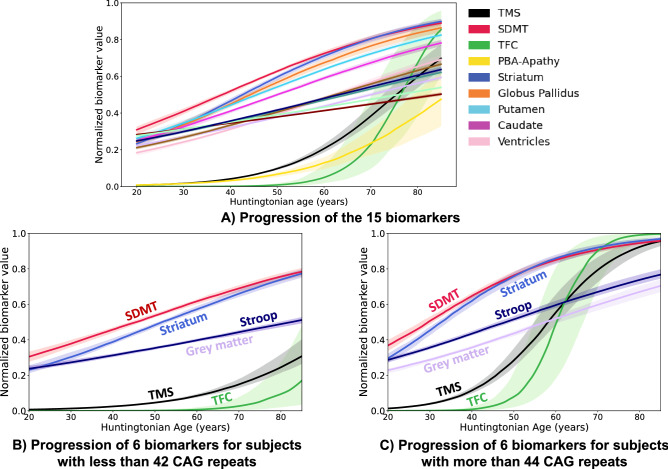
Progression of 15 biomarkers in HD (**A**) The progression estimated from all 290 participants in the TRACK-HD and TRACK-ON studies. Shaded areas correspond to the interquartile confidence intervals around the mean (central curves). Biomarkers have been rescaled so that 0 corresponds the best possible value and + 1 the maximum pathological change (see “Methods” section). Some biomarkers progress in a nearly linear fashion while others exhibit a sharp increase over time. TMS: UHDRS total motor score, Stroop: Stroop Word Reading Test, SDMT: Symbol Digit Modalities Test, TFC: UHDRS Total Functional Capacity, Striatum and grey matter: striatal and grey matter volume. (**B**,**C**) Typical progression for participants with fewer than 42 CAG repeats (**B**) or more than 44 repeats (**C**) for a selected set of 6 biomarkers.

Figure 3A shows the progression of the 15 biomarkers. Figure 3B,C show the distinct trajectories of six of these biomarkers in two subgroups: subjects with fewer than 42 CAG repeats, and those with more than 44 repeats. As expected, subjects in the group with the longest repeat lengths had earlier onset and faster progression.

**Figure 4:**
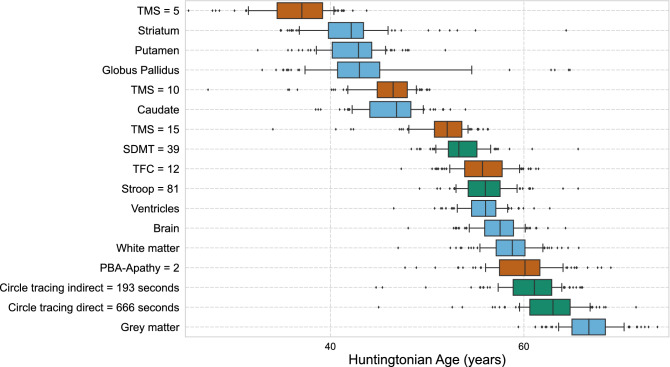
Huntington disease shows a specific temporal ordering of biomarker changes. We repeatedly calculated the age at which each biomarker diverged from control values. The central line is the median age of 100 simulations. The boxplots show the first and third quartiles, and the whiskers show the first and ninth decile. For instance, motor changes (TMS = 5) are the first detectable sign of HD at HA of 37 years, with an interquartile confidence interval from 34.5 to 39.2 years.

The 15 markers deviate from control values following the sequence shown in Fig. 4. The first biomarker to diverge from controls who do not have the HD-causing allele is TMS for a value of TMS = 5. This threshold is reached at HA of 37 years and an interquartile confidence interval [CI] from 34.5 to 39.2 years. TMS is the most powerful biomarker of early stages. We add to the Fig. 4 the HA at which further thresholds of the TMS are reached: TMS = 10 and TMS = 15.

**Figure 5:**
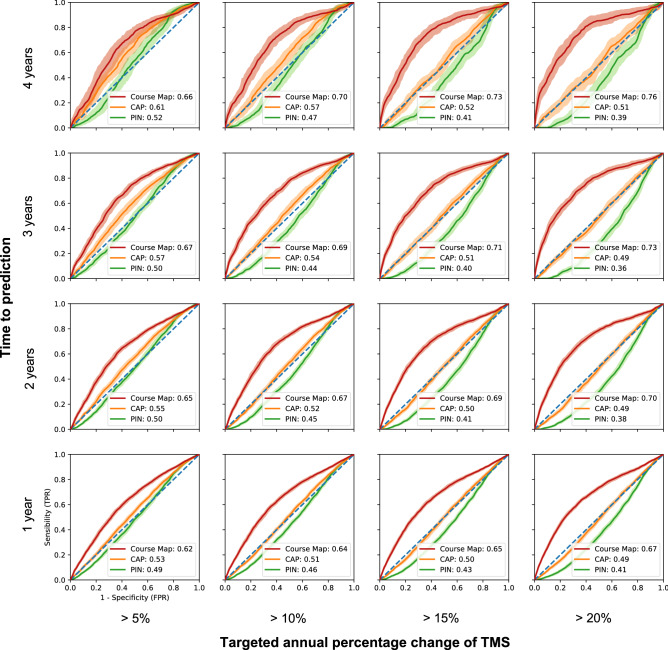
HD COURSE MAP identifies participants with TMS increase. ROC curves are shown for the detection of participants with an annual percentage change of TMS of at least 5%, 10%, 15% or 20% (in columns) in 1, 2, 3 or 4 years (in rows). Selection was made for ENROLL participants whose baseline TMS value has already diverged from controls. Three methods are compared: selection based on PIN, CAP and the TMS change from baseline that HD COURSE MAP predicts. AUCs for the three methods are reported in the legend.

Changes of basal ganglia volumes are next: striatum at 42.2 years (CI [39.8, 43.4]); putamen at 42.9 years (CI [40.2, 44.3]); globus pallidus at 43.0 years (CI [40.7, 45.1]); and caudate nucleus at 46.8 years (CI [44.1, 48.3]). These volumetric imaging markers are well suited for studies of early and possible premanifest individuals.

On the other hand, many biomarkers change only 20 years after motor onset. These late biomarkers include most clinical measurements as well as volume changes of whole brain, ventricles, total white matter, and total grey matter. These latter markers are therefore ill suited for studies of early stages.

TFC decline is a feature only of advanced HD. Despite the regulatory appeal of a functional readout, our analysis shows that TFC is an inappropriate endpoint for early-stage trials.

Overall, HD COURSE MAP succinctly summarizes the natural history of HD biomarkers. Our conclusions are consistent with current knowledge of the disease^3,15^.

### HD COURSE MAP captures differences in the trajectories of individual biomarkers and of individual participants

HD COURSE MAP not only represents the general trajectory of disease progression, but it also summarizes the variability of individual trajectories. For each subject, three parameters describe these changes (Fig. 2C). The first two parameters (time-shift τ and acceleration α) summarize differences in the timing and rate of progression. Together they reflect the Huntingtonian Age (HA). The intermarker spacing parameters (ω_j_) represent the trajectory of individual biomarker j, accounting for individual phenotypic differences at a given HA. Together with the general trajectory determined by our analysis, these parameters allowed us to describe the progression of each biomarker for each participant.

By comparing these parameters across subjects, we examined the influence of socio-demographics and genetics. Our analysis showed that some cofactors (such as longer CAG repeats) modulate HD progression, while others (such as educational level or sex) do not. On average, the disease starts 2.4 years earlier (*p* value: 5.0 × 10^−25^, CI [2.756, 1.980]), and the rate of progression is multiplied by 1.043 (*p* value = 4.3 × 10^−4^, CI [1.020, 1.067]) for every additional CAG repetition, *all other things being equal*. Increased CAG repeats also advance degradation of Stroop test (*p* = 5.5 × 10^−3^) and delays grey-matter changes (*p* = 5.4 × 10^−8^), brain atrophy (*p* = 4.9 × 10^−4^) and ventricles volumes (*p* = 5.1 × 10^−4^) after normalizing age at onset and pace of progression. See supplementary Figure 1 for further results.

### HD COURSE MAP forecasts individual biomarkers up to 5 years in advance.

Using baseline data, we placed each new mutation carrier on the HD COURSE MAP by estimating the individual parameters τ, α, and ω, that best describe the subject’s biomarkers. For each participant from ENROLL-HD with TMS greater or equal to 5 (over 124) at baseline, we then placed the subject’s progression along the trajectory of each biomarker, and we compared these forecasts to subsequent measurements, 1, 2, 3, 4 and 5 years later.

We repeated this procedure for SDMT ≤ 38 (/110), PBA-Apathy ≥ 3 (/8), TFC ≤ 12 (/13), and Stroop < 81 at baseline. The mean absolute error (MAE) of predicted TMS is of 7.29 points (out of 124) at 1 year and 9.8 points at 5 years, an error of 5.8% and 7.9% respectively.

Similarly, we used TRACK-HD participants for a study of volumetric changes in subcortical structures. These forecasts were remarkably precise, with mean absolute errors at 4 years for caudate nucleus 2.5 × 10^−4^ and for putamen 2.0 × 10^−4^. See Supplementary tables for all forecast results.

We compared our predictions to two others based on other assumptions: (1) that the measurements do not change from baseline, and (2) that changes occur linearly, with a slope that depends on age and the number of CAG repeats.

Because our subjects are at early stages in disease progression, the no-change assumption makes good short-term predictions. By contrast, HD COURSE MAP better forecast TMS, SDMT and TFC at 2 years and more. The mean prediction of TMS, SDMT and Stroop ranges between 6 and 8%. Predictions for PBA-Apathy (resp. TFC) are less good, with errors of 1.1 point out of 8 (resp. 2.15 out of 13), which corresponds to 14% (resp. 16.5%). The linear model, taking account of age and number of CAG repeats, does no better than the no-change prediction.

### HD COURSE MAP can select participants before significant disease progression.

We next evaluated the ability of HD COURSE MAP to select participants whose biomarkers would change during a given period. As before, we started with participants whose TMS was greater than 5, with the goal of selecting those whose TMS would increase annually by more than 5%, 10%, 15% or 20%. Figure 5 shows the Receiver Operating Characteristic (ROC) curve for the three selection methods: CAP value at baseline, PIN value at baseline, and TMS change from baseline that HD COURSE MAP predicts. Supplementary Figures 2– 6 show results for the other biomarkers. These ROC curves demonstrate that HD COURSE MAP is better at identifying subjects whose markers will advance: the area under its curve is greater than those of the other two methods—for all time points and for all markers, excepting TFC.

In particular, HD COURSE MAP is better for the TMS, Stroop and SDMT, consistent with the predictive errors. For earlier-stage participants (0 < TMS < 5), however, CAP and PIN give a better selection at 3 and 4 years. Still, for ENROLL-HD participants with TMS > 5, HD COURSE MAP is best at forecasting individual trajectories.

### HD COURSE MAP can design more powered clinical trials

Our ability to predict the trajectories of outcome measures for each mutation carrier can greatly improve subject selection for clinical trials. Knowledge of the temporal ordering of biomarker allows us to select outcome measures that progress most rapidly at the stage targeted by the drug (Fig. 4). Trialists will then be able to select participants close to the time when selected outcome measures will change. This approach would reduce the fraction of subjects in whom an outcome measure is unlikely to change during a trial.

**Figure 6:**
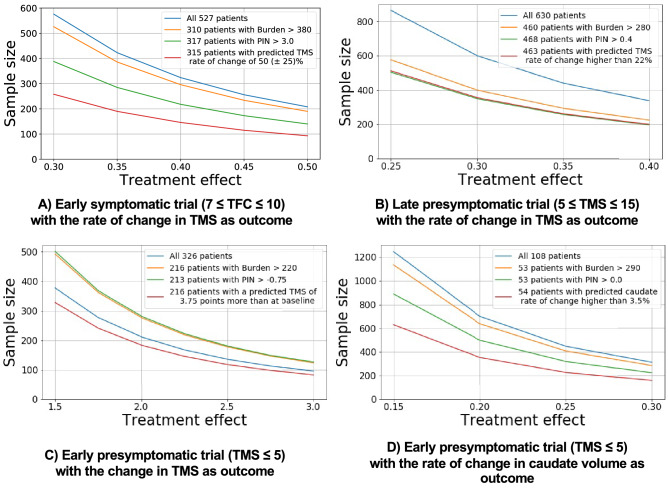
HD COURSE MAP reduces the number of necessary participants compared to PIN and CAP. Each panel represents a simulated trial on a particular disease stage with different outcome measures. Blue curves represent every participant meeting the prespecified inclusion criteria. Orange, green and red curves represent participants selected based on the burden score, the PIN score or HD COURSE MAP respectively. The experiments in panels (**A**–**C**) use ENROLL participants. Experiment in (**D**) use TRACK participants.

To test the ability of HD COURSE MAP to help clinical trial design, we simulated four clinical trials (Figs. 1 and 6), each centered on a different stage of disease development, from premanifest to symptomatic (Table 2). Because HD COURSE MAP selects participants with greater predicted changes and with smaller variance, the required sample size was lower than with the PIN or the CAP (Fig. 6).

**Table 2:** Trial designs to compare selection procedures and sample sizes (Fig. 6).

	Premanifest or very early stages defined by motor score (maximum worse value TMS = 124)			Early affected based on functional (maximum best value TFC = 13)
Inclusion criteria	0 ≤ TMS ≤ 5	0 ≤ TMS ≤ 10	5 ≤ TMS ≤ 15	7 ≤ TFC ≤ 10
Trial duration	4 years	2 years	3 years	3 years
Outcome measure	Change of absolute TMS	Rate of change of normalized caudate nucleus volume	Rate of change of TMS	Rate of change of TMS
Treatment effect	1.5–3.0 points less change in the TMS	15–30% less change in the caudate atrophy rate	25–40% less in the TMS rate of change	30–50% less in the TMS rate of change
HD COURSE MAP	Estimated on TFC, TMS, SDMT & STROOP			Estimated on TMS, and caudate nucleus and putamen volumes
Experiments	319 predictions based on 152 patients from TRACK HD/ON (using cross-validation)	HD Course map estimated on TRACK-HD/ON. 330 predictions on ENROLL	HD Course map estimated on TRACK-HD/ON. 665 predictions on ENROLL	HD Course map estimated on TRACK-HD/ON. 527 predictions on ENROLL

Simulations target different disease stages, trial durations and outcome measures. Three designs aim to reproduce the ones of the Crest-HD, Pride-HD and 2Care-HD trials^24–26^.

Our first simulation started with 527 ENROLL participants in early symptomatic stages, with a baseline functional score (TFC) between 7 and 10. We simulated a trial whose endpoint was a 40% reduction in the rate of TMS change over the 3 years of the study. All three selection methods decreased the number of required participants. HD COURSE MAP was the most effective, reducing sample size from 527 to 150; PIN reduced the number of required subjects to 220, and CAP to 300.

The second simulation sought a 30% reduction TMS change after 3 years, beginning with 630 ENROLL participants with TMS between 5 and 15. In this case, PIN and HD COURSE MAP had similar performance, yielding a sample size of 360 while CAP required a sample size of 400.

The third simulation started with 326 ENROLL participants who were premanifest according to TMS, with TMS < 5. The endpoint of this simulated trial was a 3-point decrease in TMS at the end of a 4-year trial. Although PIN and CAP better identified participants in this cohort (Supplementary Figure 6), we found a greater heterogeneity in TMS changes, leading to a greater sample size than initially stipulated. HD COURSE MAP required a sample size of 85 participants, compared to 100 without any selection, yielding a 15% reduction for this most challenging situation.

Finally, we simulated an imaging-only trial with TMS < 10, with the 2-year endpoint a 25% decline in the rate of caudate atrophy. Because ENROLL-HD did not include imaging data, we used 108 TRACK HD and TRACK-ON participants in a cross-validation procedure. HD COURSE MAP allowed to calculate a sample size of 210, whereas PIN required 320 subjects and CAP 400. Here our method reduced the number of needed participants by 48% and 34%.

## Discussion

We applied HD COURSE MAP, a machine learning technique, to capture the detailed progression of clinical, cognitive, and imaging biomarkers during the course of HD. Once trained, our method was able to forecast biomarker values for a new participant from a single baseline visit. HD COURSE MAP, applied to clinical trials, would allow to identify individuals most likely to progress and enrich the tested trial population.

We have used data from TRACK-HD and TRACK-ON, two carefully controlled studies across four experienced research sites with low variability between sites^15–19^. The low variability between sites also gave us confidence in the repeated measurements in the 7 years spanned by the two follow up studies. Training with only 290 subjects from TRACK-HD and TRACK-ON was sufficient to forecast detailed outcomes for 11,510 participants in ENROLL-HD excluding the imaging since it was not available from the ENROLL-HD data.

In the early 2000s, survival analysis was mainly used to estimate age at onset, based on the number of CAG-repeats in the pathological expansion^7,20^. TRACK-HD used Langbehn’s model to stratify expansion carriers, dividing subjects into two groups, based on a predicted time to disease below or above 10.8 years. Also using CAG repeat number and age as the main determinants, Zhang et al. divided expansion carriers into three groups with differing onset probabilities^8^. More recently, Paulsen et al. developed a sample enrichment recruitment method to facilitate clinical trial in pre-manifest mutation carriers^12^. Polosecki et al. proposed a stratification of disease progression, based on resting-state fMRI^21^. These recent models have pursued the same aim of modelling disease progression and made significant contributions. Our model continues in the same direction and offers additional possibilities. We provide a digital brain twin that can predict at the individual level the value of imaging and cognitive markers at up to 4 years in the future. We summarized in this study the progression of HD for several biomarkers and used CAG repeat number only as a covariate rather than the main determinant of disease progression. In addition, the large variability for a given CAG repeat size with age at onset or progression is well described^22,23^. Recent studies showed that genetic modifiers and somatic expansion with age influences the phenotype^24,25^.

Other researchers have explored the relationship between biomarker assessment and disease progression. Aylward and colleagues demonstrated that decreased striatal volume increased the probability of diagnosis, and Beglinger et al. showed how motor, cognitive, and depression scores from the UHDRS predicted functional decline^26,27^. Langbehn et al. explored the influence of CAG repeat length on biomarker progression. As in our analysis, increased CAG repeats led to earlier onset and faster progression^28^. The main difference with the method presented in this study is the fact that the cited studies are population-based and do not predict the individual timings and trajectories. They indubitably provide valuable insights on the determinants of disease progression, but because they are population-based, they do not allow for the scaling back to individuals’ progression and personalised prediction.

We have demonstrated the possible utility of our method by comparing its ability to reduce the number of participants in simulated clinical trials with the one of the current selection methods. We made no reference to the participants being labelled as pre-manifest or manifest because our model generates a continuous trajectory of the disease on which any patient can be precisely positioned based on the baseline biomarker profile.

Trials usually control for the disease stage at inclusion, reflected by thresholds on clinical scales (e.g. TMS or TFC) or measures (e.g. PIN). But controlling the disease stage does not imply controlling the progression rate. From the same disease stage, participants still exhibit heterogeneous disease progression and presentation, a fact that inflates the sample size required to show a given treatment effect. By contrast, HD COURSE MAP select participants based on the predicted progression of the outcome, thus decreasing the variance of the outcome, increasing the effect size (expressed as a percentage of the outcome change), and therefore the required sample size.

HD COURSE MAP also informs about the choice of the outcome according to the targeted disease stage. For instance, it shows that TFC decline is a feature of manifest HD only. We therefore recommend not using TFC as an endpoint for trials with premanifest individuals, since TFC flattens until disease onset. By contrast, TFC was used successfully as outcome for trials targeting manifest HD participants.

The concentrations of neurofilament light chain in blood and in CSF have emerged as useful biomarkers for axonal damage prior to clinical changes^29^. The same holds true for promising neuroimaging polymarker including structural and functional modalities in the premanifest stages^30^. In the dataset used we did not include these biomarkers but they might further enhance the predictive value of HD COURSE MAP and this could be actual limitation.

Given the tremendous personal and physical engagement required for both participants and researchers in a clinical trial, minimizing the number of required subjects remains an important goal for HD clinical research. We showed that machine learning for Huntington disease can help accomplish this goal by identifying individuals whose condition measured by biomarkers progressed most rapidly. In fact, participants at baseline for whom we forecast little change of the outcome would not need to be followed annually, thus limiting the burden. Machine learning has also proved more useful than conventional approaches in other progressive conditions^31,32^.

## Methods

### Data sets

The study used data from two multicentre observational studies: TRACK-HD and TRACK-ON^15–19^. These studies measured motor, cognitive, and neuropsychological biomarkers. They also determined the volumes of several brain structures^33,34^. The purpose of these studies was to identify endpoints sensitive to disease progression, starting well before anticipated clinical onset. To validate our prediction method in an independent cohort, we used data from ENROLL-HD, a global clinical research platform designed to facilitate clinical research in HD.

We obtained data from the portal https://www.enroll-hd.org/. Informed consent forms were signed by the participants or a legally acceptable representative (https://www.enroll-hd.org/enrollhd_documents/Enroll-HD-Protocol-1.0.pdf). The use of these data for carrying out the presented work has been approved by the following ethical review committees: comité de protection des personnes (CPP) Ile de France III Hôpital Tarnier-Cochin under reference 3188 (ENROLL-HD) and comité de protection des personnes (CPP) Ile de France IV groupe hospitalier Pitié-Salpêtrière under reference 6407 (TRACK-HD and TRACK-ON). The research has been performed in accordance with the Declaration of Helsinki. The research was performed in accordance with relevant guidelines and regulations.

### Feature selection and processing

The TRACK-HD and TRACK-ON cohorts report various clinical, cognitive, and imaging measurements from which we kept the variables that meet the following conditions. Variables must show a significant difference between mutation-carriers and non-mutation carriers and their rate of change should be significantly different from 0 (Mann–Withney U test *p* value smaller than 0.005 corrected for multiple comparisons). In addition, to avoid variables with a non-monotonic progression, we removed variables which presented more than 40% of patients reverting and those that were not having at least 55% of patients with a worsening or stability over time.

We considered that all biomarkers would increase during the disease progression. For biomarkers that decrease with progression (such as volumetric measurements), we reversed their temporal profile.

We normalized each reading to a scale of 0 to + 1. For bounded assessments, we normalized them based on their theoretical maximum and minimum values. For unbounded measurements such as ones measured in seconds or imaging derived volumes, we calculated Z-scores based on their distribution in control individuals from TRACK-HD and TRACK-ON. The normalisation procedure does not use any data from mutation carriers.

In each case, most temporal profiles start at values near 0 (control value), increasing to 1 (abnormal values) with disease progression. Some features start with values greater than zero, even in controls.

We then apply this normalization procedure to the measurements of the participants in the ENROLL-HD study.

### Multimodal digital model of disease progression (HD COURSE MAP)

HD COURSE MAP is built on a new statistical learning technique named disease course mapping^13,14,35,36^. The method has two goals: (1) to model the long-term changes of clinical and imaging biomarkers from the repeated observation of multiple subjects at various disease stages; (2) to predict the future values of these biomarkers from measurements at the entry of each new participant.

The progression of the measurements is broken down according to three parameters (Fig. 2).

1. time-shift τ_i_, the temporal onset of disease progression for all measurements in subject i; τ_i_ is negative for earlier-than-average progression and positive for later-than-average progression,
2. acceleration α_i_, the change in the rate of progression of subject i; α_i_ = 1 means that, for that subject, all the biomarker trajectories match the idealized curves after adjusting the timing with τ_i_ and ω_i_. When α_i_ > 1, it takes less time for the same changes to occur, e.g. α_i_ = 2 means that it will twice as fast as average for that subject to undergo a given change in a biomarker trajectory,
3. intermarker spacing ω_ij_, the temporal ordering of biomarker j with respect to other biomarkers for subject i. The sign of ω_ij_, indicates whether a single feature in a particular subject exhibits earlier or later changes relative to other features (after normalizing the age at onset τ_i_ and pace of progression α_i_): ω_ij_ < 0 means earlier degradation, and ω_ij_ > 0 means later.

The first two parameters allow us to map the chronological age of the participant onto his Huntingtonian Age (HA). For each single visit, HA is obtained using an affine transformation of the chronological age. While HA increases with disease progression, it does not directly correspond to the subject’s chronological age. Two different subjects are observed at the same disease stage: they will have the same HA but a different chronological age. If they have a different acceleration factor, it will take more or less time to them to gain 1 year of HA. On average, 1 year in the HA axis corresponds to 1 year in chronological age.

At the population level, HD COURSE MAP describes the progression of the endpoints with a set of logistic curves indexed by HA. We fit this set of curves to the repeated measurements of a participant by estimating the individual parameters (τ_i_, α_i_, and ω_ij_) for each individual. Each mutation carrier has a single τ, a single α, and one ω for each biomarker (15 in this analysis). We determine the shape and position of the logistic curves so that mean value of τ_i_ across all subjects is 0, the mean value of the logarithm of α_i_ is 0, and the mean value of each ω_ij_ is 0. All in one, the estimation of the disease course map takes the form of a non-linear Bayesian mixed-effects model where the shape and position of the logistics curves represent the fixed effects and the subject’s parameters (τ_i_, α_i_, and ω_ij_) the random effects. We iteratively performed joint estimations of these population parameters with the individual ones to minimize errors between the predicted and observed measurements. The result is called HD COURSE MAP.

We use then HD COURSE MAP to predict the disease trajectory from the baseline measurements of a new participant. We estimate the individual parameters for the subject and then transform the model to fit the measurements. (Fig. 2D). This process accomplishes two goals: (1) characterization of subjects according to values of τ, α and ω (Fig. 2B,C); and (2) forecast of future biomarker values (Fig. 2D). Individual parameters are estimated by maximizing the likelihood of the new participant’s data using the estimated mean and variance of these parameters in the training population as priors.

Such priors allow us to estimate the most probable pace of progression even from baseline measurements only. The prediction of the trajectory is entirely driven by the series of endpoints at a given age. These series of measurements contain a signature that is predictive of the progression. In particular, we do not use the number of CAG repeats to predict the progression of the endpoints. We test a posteriori that the estimated pace of progression is highly correlated with the number of CAG repeats as a validation measure.

We use the publicly available software Leaspy to estimate the model parameters from a longitudinal data set and fit the trained model to new data. The software is publicly available at: https://gitlab.com/icm-institute/aramislab/leaspy/.

### Experimental design

We calibrated HD COURSE MAP from TRACK-HD and TRACK-ON data with a minimum of two visits per participant. Confidence interval for population parameters were estimated over 100 bootstrap runs that each included only 80% of the participants. We compared the mean progression of each feature in terms of HA to predefined thresholds, thereby revealing the temporal ordering of the deviation of the biomarkers from their control values. For that purpose, we used the point where the biomarker exceeded the 95th percentile of the control distribution. We then performed multivariate regression of each individual parameter of the model against five cofactors: sex, number of CAG repeats, level of education, number of visits and handedness. To estimate confidence intervals on the *p* values, we bootstrapped the model 100 times and ran 100 multivariate analyses. The reported *p* values correspond to the median over the runs.

To use ENROLL-HD as external validation cohort, we re-estimated HD COURSE MAP from the same participants in TRACK studies but using only the biomarkers that are in common between TRACK and ENROLL-HD (see Fig. 1). We use then baseline data from ENROLL-HD participants and forecast their data at follow-up visits after 1, 2, 3, and 4 years. The prediction of a given biomarker is made on ENROLL-HD participants for whom the baseline value of this biomarker already deviates from control.

We defined 16 target groups (4 times 4) within these participants: the participants for whom the annual percentage change of the biomarker will progress by 5%, 10%, 15% and 20% in 1, 2, 3 and 4 years. We select these participants based on thresholds on the CAP, PIN, and the changes in biomarkers predicted by HD COURSE MAP and build the corresponding ROC curve and compute the AUC. We used PIN instead of MRS as it was conceived as an extension and improvement of the MRS given external validation cohorts^10^.

We adapted the procedure for the volumetric measurements of deep brain structures. We did the prediction and selection on TRACK participants as ENROLL data do not contain imaging. We used therefore a cross-validation strategy, training HD COURSE MAP on 80% of the participants and evaluating forecasts on the remaining 20% (Fig. 1).

We demonstrate the value of our method by applying it to identify the potential participants most likely to progress during clinical trials targeting early HD patients. We simulate clinical trials with different durations, theoretical treatment effects, inclusion criteria, endpoints and disease stages (Table 2). Trial simulation designs were inspired by the past clinical trials, in particular Crest-D, Pride-HD and 2Care-HD^37–39^.

We define disease stage based on arbitrary thresholds on TMS or TFC as in real clinical trials. After choosing the disease stage, the corresponding outcome and duration for each trial, we further selected subjects by choosing thresholds predicted by each of the three methods: CAP, PIN and HD COURSE MAP. In all cases, the chosen thresholds select 50% to 70% of the participants. We ensure the number of selected participants to be the same for all three methods for a fair comparison. For these simulations, we used HD COURSE MAP calibrated on 4 biomarkers only to avoid requesting too many data from participants at screening (Fig. 1).

We then simulated the necessary sample size to detect a drug effect (the treatment effect) using the empirical distribution of the outcome measure within each selected group. For µ (resp. σ) the mean (resp. standard deviation) of the empirical distribution of the outcome in the selected population, we compute the sample size (for α = 0.05 and β = 90%) that enables to detect an effect size equal to T*µ/σ for a range of treatment effect size T, similarly to Langbehn et al.^11^.

## Supplementary Information

Supplementary Information.

## Data Availability

The datasets analyzed during the current study are available in the controlled-access repository: https://enroll-hd.org/. We obtained permissions to use these data sets from the repository. Results have been obtained by the use of the open-source software Leaspy, publicly available at: https://gitlab.com/icm-institute/aramislab/leaspy/.
