# Supplementary material for: Forecasting individual progression trajectories in Huntington disease enables more powered clinical trials
Source: Sci Rep. 2022 Nov 7;12:18928. doi: 10.1038/s41598-022-18848-8 (PMC9640581; doi:10.1038/s41598-022-18848-8)
Supplement: Supplementary file 1 — Supplementary Information. [file 41598_2022_18848_MOESM1_ESM.pdf]

Forecasting individual progression trajectories in Huntington disease – application to clinical trials

Igor Koval, Thomas Dighiero-Brecht, Allan J. Tobin, Sarah J Tabrizi, Rachael I Scahill, Stanley Durrleman, Alexandra Durr

SUPPLEMENTARY MATERIAL

Supplementary Tables: Forecasts of participant's biomarkers 1, 2, 3, 4 and 5 years after their baseline measurements. We report the mean absolute error (MAE) for three prediction methods: HD Course Map, error when predicting no change, and a linear regression model using age and CAG as predictor. Confidence intervals (5th and 95th percentiles) are computed with a bootstrap method (500 bootstrap runs with 70% of the predictions each, including replacement). Results in bold are significantly better than the other two.

a) MAE for the Total Motor Score, Symbol Digit Modalities Test, Total Functional Capacity, STROOP and PBA-Apathy of ENROLL participants. HD Course Map was trained on TRACK participants only.

Total Motor Score

	1 year	2 years	3 years	4 years	5 years
**HD Course Map**	7.29 [7.17, 7.41]	**8.30 [8.14, 8.46]**	**9.23 [8.99, 9.51]**	**9.89 [9.41, 10.38]**	**9.81 [8.61, 10.93]**
**No Change**	7.33 [7.23, 7.46]	9.47 [9.25, 9.65]	11.78 [11.43, 12.12]	13.45 [12.79, 14.15]	14.30 [12.64, 15.86]
**Linear**	16.08 [15.85, 16.31]	16.09 [15.80, 16.38]	16.11 [15.69, 16.50]	15.52 [14.78, 16.30]	14.39 [13.06, 15.59]

Symbol Digit Modalities Test

	1 year	2 years	3 years	4 years	5 years
**HD Course Map**	4.62 [4.53, 4.71]	**4.85 [4.74, 4.98]**	**5.01 [4.84, 5.21]**	**5.64 [5.26, 6.02]**	**6.35 [5.45, 7.33]**
**No Change**	**4.25 [4.16, 4.34]**	5.24 [5.10, 5.37]	6.42 [6.21, 6.64]	7.65 [7.23, 8.05]	8.95 [7.96, 10.11]
**Linear**	7.94 [7.82, 8.08]	7.90 [7.72, 8.09]	7.75 [7.52, 8.00]	7.77 [7.35, 8.18]	8.31 [7.32, 9.34]

Total Functional Capacity

	1 year	2 years	3 years	4 years	5 years
**HD Course Map**	1.24 [1.21, 1.26]	**1.52 [1.48, 1.56]**	**1.74 [1.68, 1.80]**	**2.02 [1.91, 2.15]**	**2.15 [1.88, 2.41]**
**No Change**	**1.20 [1.17, 1.23]**	1.61 [1.57, 1.65]	2.01 [1.95, 2.09]	2.33 [2.19, 2.46]	2.58 [2.31, 2.89]
**Linear**	2.66 [2.62, 2.70]	2.64 [2.59, 2.68]	2.62 [2.54, 2.69]	2.59 [2.47, 2.71]	2.54 [2.29, 2.78]

STROOP

	1 year	2 years	3 years	4 years	5 years
**HD Course Map**	10.66 [10.47, 10.86]	10.51 [10.27, 10.77]	**10.59 [10.24, 10.96]**	**11.20 [10.54, 11.85]**	**12.68 [11.19, 14.27]**
**No Change**	**8.67 [8.50, 8.84]**	10.57 [10.30, 10.81]	12.48 [12.09, 12.90]	14.56 [13.73, 15.24]	15.69 [13.97, 17.43]
**Linear**	14.78 [14.55, 15.04]	14.64 [14.35, 14.97]	14.44 [14.04, 14.83]	14.57 [13.86, 15.40]	15.11 [13.48, 16.89]

PBA-Apathy

	1 year	2 years	3 years	4 years	5 years
**HD Course Map**	1.18 [1.16, 1.21]	1.08 [1.06, 1.11]	1.20 [1.16, 1.24]	1.29 [1.21, 1.36]	1.12 [0.96, 1.27]
**No Change**	**0.99 [0.97, 1.01]**	1.10 [1.07, 1.13]	1.19 [1.15, 1.24]	1.29 [1.21, 1.37]	1.20 [1.02, 1.39]
**Linear**	1.14 [1.12, 1.15]	1.19 [1.17, 1.21]	1.22 [1.19, 1.25]	1.28 [1.22, 1.33]	1.16 [1.04, 1.27]

b) MAE for the striatum, caudate nucleus, globus pallidus and putamen volumes of TRACK participants (all normalized by participants intracranial volume). HD Course Map was trained and tested on TRACK participants in a cross-validation setting. Forecasts durations vary according to data availability.

Striatum volume, normalized by intracranial volume (x 10^4)

	1 year	2 years
**HD Course Map**	**1.24 [0.98, 1.53]**	**1.44 [0.92, 2.04]**
**No Change**	1.69 [1.40, 1.97]	2.71 [2.10, 3.36]
**Linear**	3.80 [3.06, 4.69]	4.18 [3.01, 5.24]

Caudate nucleus volume, normalized by intracranial volume (x 10^4)

	1 year	2 years	3 years	4 years
**HD Course Map**	**3.24 [2.94, 3.54]**	**3.87 [3.44, 4.32]**	**2.46 [1.81, 3.13]**	**2.53 [2.00, 3.21]**
**No Change**	3.84 [3.51, 4.18]	5.57 [5.09, 6.09]	2.48 [1.81, 3.21]	4.47 [3.59, 5.36]
**Linear**	5.61 [5.14, 6.15]	5.82 [5.08, 6.53]	5.29 [3.97, 7.09]	4.63 [3.34, 5.96]

Globus Pallidus volume, normalized by intracranial volume (x 10^4)

	1 year	2 years
**HD Course Map**	2.96 [2.66, 3.31]	3.06 [2.54, 3.66]
**No Change**	**1.78 [1.60, 1.98]**	2.55 [2.19, 2.95]
**Linear**	6.89 [6.20, 7.69]	6.88 [5.70, 7.86]

Putamen volume, normalized by intracranial volume (x 10^4)

	1 year	2 years	3 years	4 years
**HD Course Map**	**1.00 [0.93, 1.08]**	**1.38 [1.28, 1.49]**	**1.71 [1.56, 1.90]**	2.04 [1.75, 2.32]
**No Change**	1.30 [1.22, 1.37]	1.96 [1.83, 2.09]	2.33 [2.12, 2.56]	2.08 [1.81, 2.39]
**Linear**	5.97 [5.58, 6.37]	6.00 [5.51, 6.51]	6.02 [5.42, 6.72]	5.38 [4.66, 6.14]

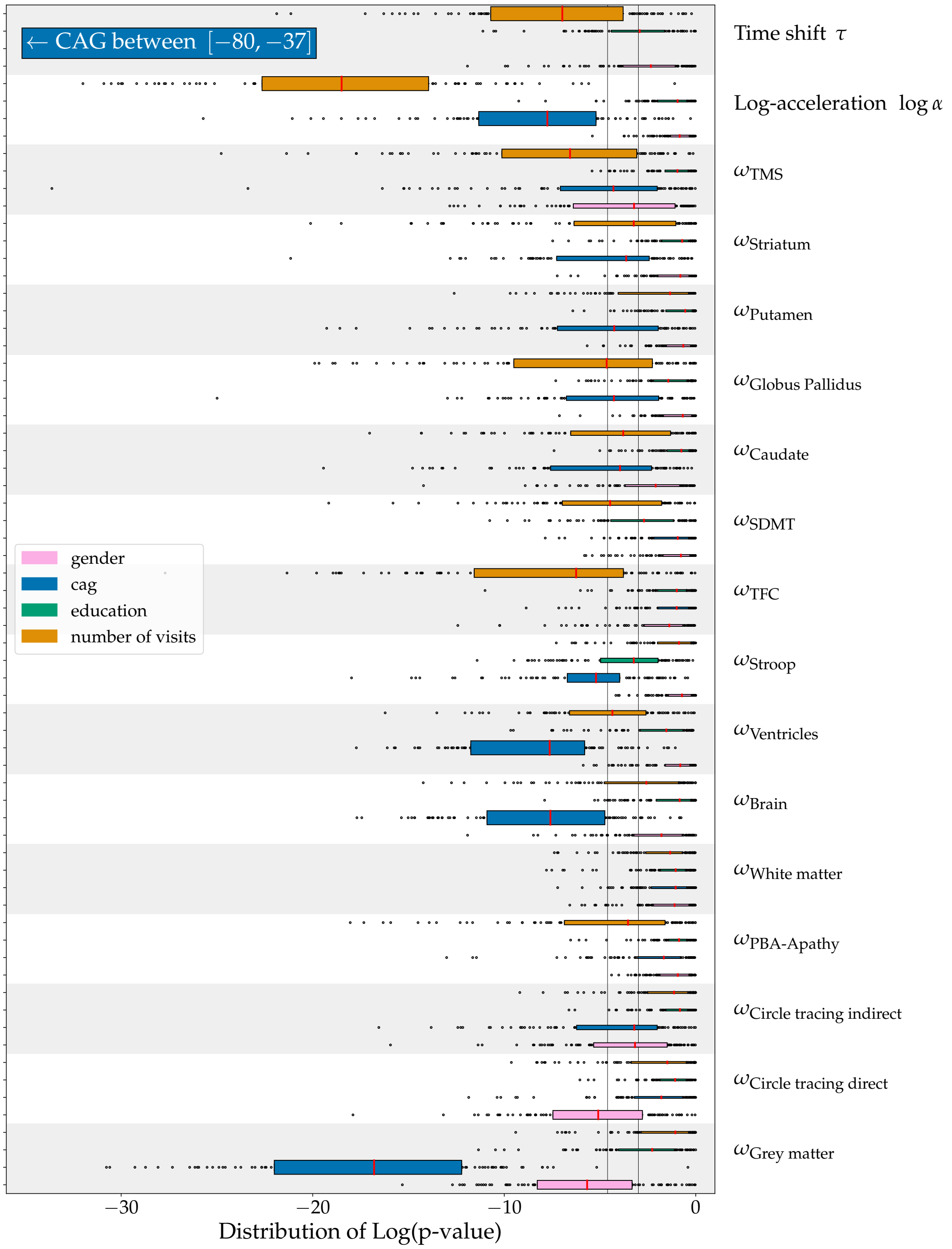

Supplementary Figure 1: Association of individual parameters with genetics and socio-demographics. Multivariate regression of each model parameter (right) against five covariates: sex, number of CAG repeats, level of education, number of visits and handedness. To estimate confidence intervals on the p-values, we bootstrapped the model 100 times and ran 100 multivariate analyses. The reported p-values correspond to the median over the runs. The two black vertical bars correspond to the 0.05 and 0.01 thresholds. Neither education level nor sex influences the speed of progression or the age at onset. Increased CAG repeats advance Stroop abnormality ($p=5.5\ 10^{-3}$) and delays grey-matter changes ($p = 5.4\ 10^{-8}$), brain atrophy ($p = 4.9\ 10^{-4}$) and ventricles volumes ($p = 5.1\ 10^{-4}$) compared to the average progression. Sex influences on circle tracing and grey matter needs further investigation. The number of visits influenced every clinical variable but the Stroop test, as well as all the volumes of basal ganglia structures, but it had no influence on grey and white matter volume or on ventricular volume. The number of visits as a covariate reflects a standard bias in cohorts: subjects with more visits are those that can be followed during years, which is usually related to a slow disease progression - fast progressors are less likely to be evaluated for long periods of time because of death, physical or mental disabilities.

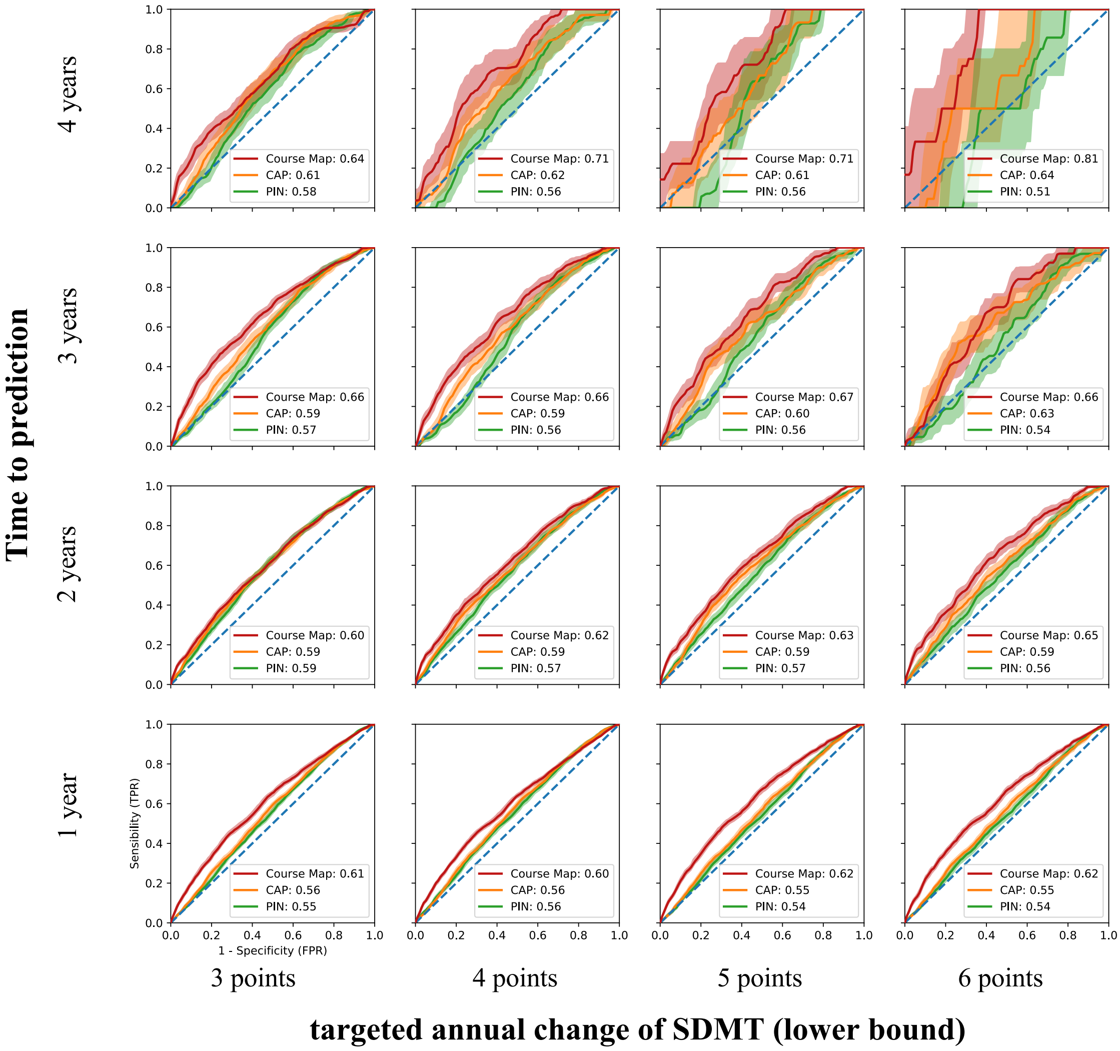

Supplementary Figure 2: HD COURSE MAP identifies participants experiencing changes of SDMT. ROC curves are shown for the detection of participants with an annual change of SDMT of at least 3, 4, 5 or 6 points (in columns) in 1, 2, 3 or 4 years (in rows). Selection was made for ENROLL participants whose baseline SDMT value has already diverged from controls. Three methods are compared: selection based on the PIN, Burden and the SDMT change from baseline that HD COURSE MAP predicts. AUCs for the three methods are reported in the legend.

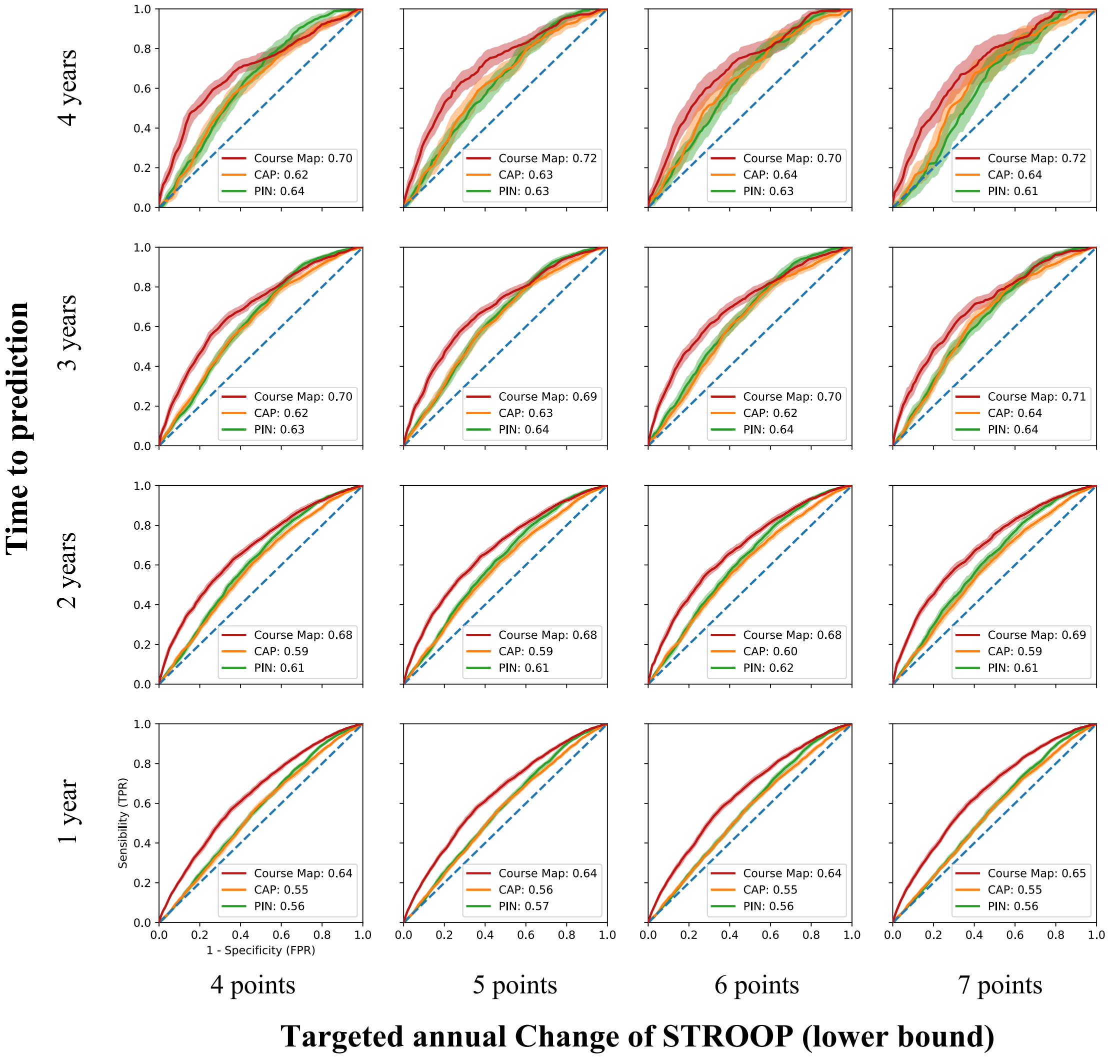

Supplementary Figure 3: HD COURSE MAP identifies participants experiencing changes of Stroop reading test. ROC curves are shown for the detection of participants with an annual change of at least 4, 5, 6 or 7 points (in columns) in 1, 2, 3 or 4 years (in rows). Selection was made for ENROLL participants whose baseline value of the Stroop test has already diverged from controls. Three methods are compared: selection based on the PIN, Burden and the SDMT change from baseline that HD COURSE MAP predicts. AUCs for the three methods are reported in the legend.

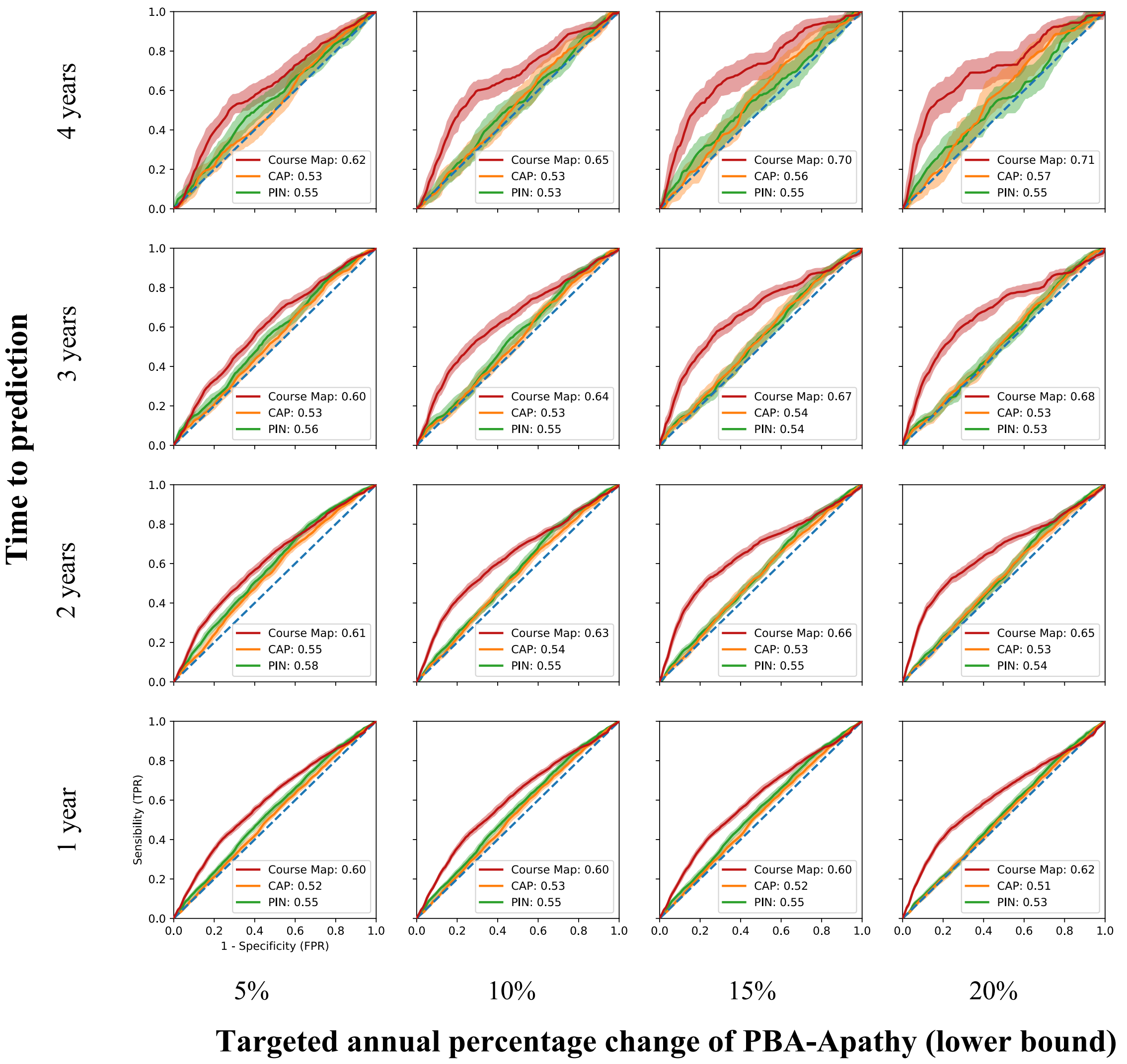

Supplementary Figure 4: HD COURSE MAP identifies participants experiencing changes in PBA-Apathy scale. ROC curves are shown for the detection of participants with an annual percentage change of at least 5%, 10%, 15% or 20% points (in columns) in 1, 2, 3 or 4 years (in rows). Selection was made for ENROLL participants whose baseline PBA-Apathy assessment has already diverged from controls. Three methods are compared: selection based on the PIN, Burden and the SDMT change from baseline that HD COURSE MAP predicts. AUCs for the three methods are reported in the legend.

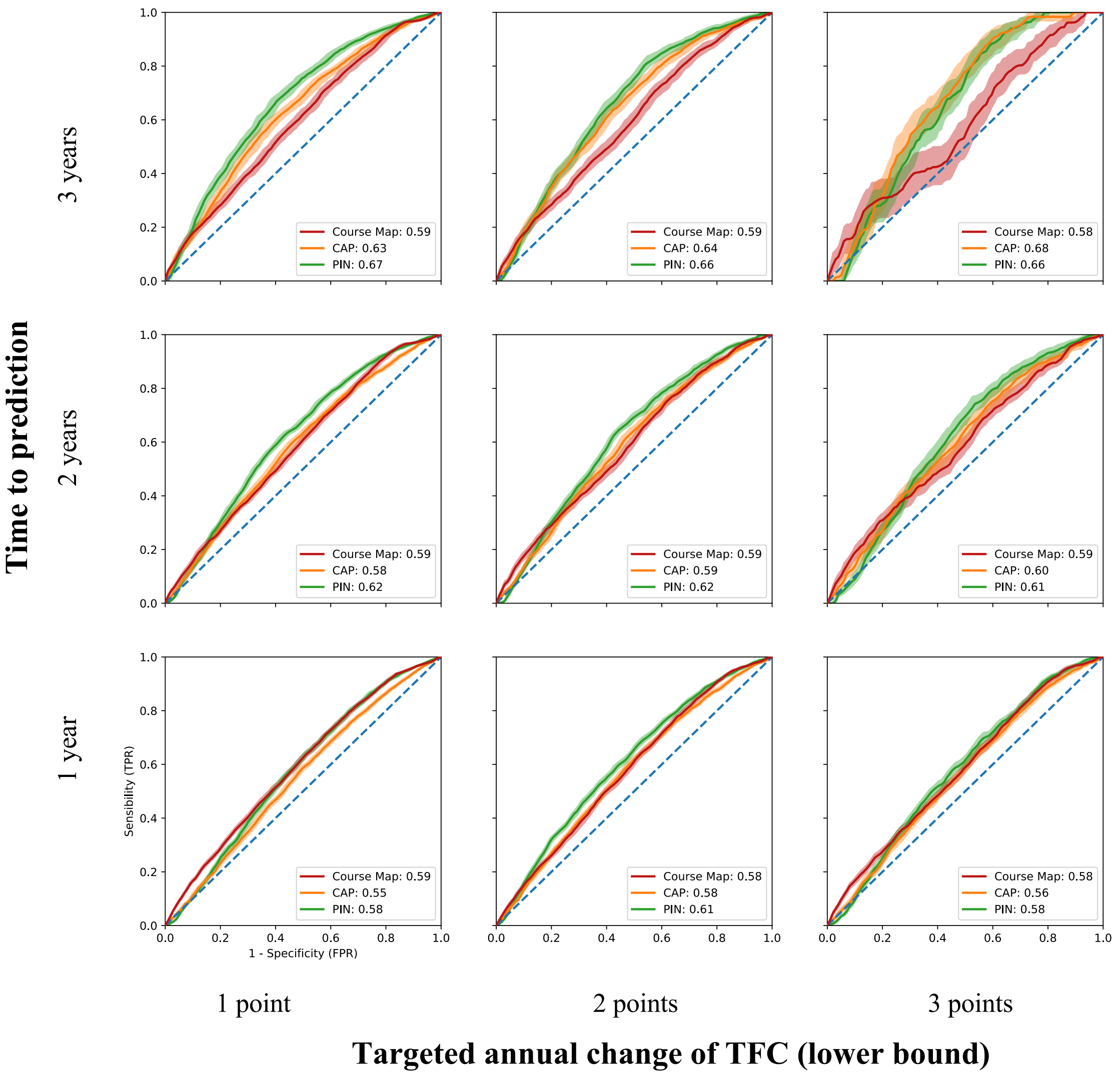

Supplementary Figure 5: HD COURSE MAP identifies participants experiencing changes of TFC. ROC curves are shown for the detection of participants with an annual change of TFC of at least 1, 2 or 3 points (in columns) in 1, 2, 3 or 4 years (in rows). Selection was made for ENROLL participants whose baseline TFC value has already diverged from controls. Three methods are compared: selection based on the PIN, Burden and the SDMT change from baseline that HD COURSE MAP predicts. AUCs for the three methods are reported in the legend.

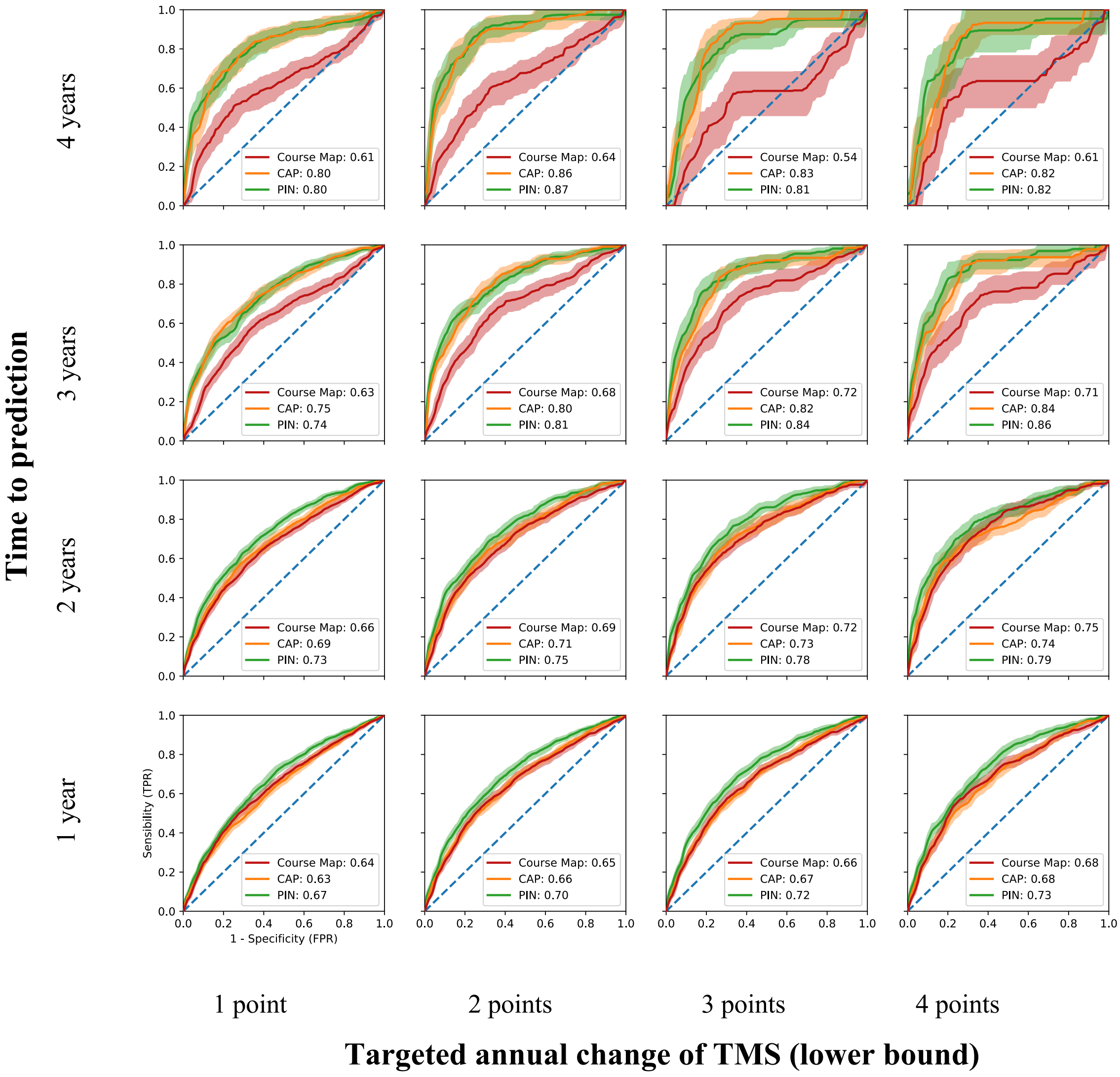

Supplementary Figure 6: HD COURSE MAP identifies participants experiencing changes of TMS from a baseline value ranging from 0 to 5. ROC curves are shown for the detection of participants with an annual change of TMS of at least 1, 2, 3 or 4 points (in columns) in 1, 2, 3 or 4 years (in rows). Selection was made for ENROLL participants whose baseline TMS value was smaller than 5. Three methods are compared: selection based on the PIN, Burden and the SDMT change from baseline that HD COURSE MAP predicts. AUCs for the three methods are reported in the legend.
